# 2025 AAC Extraordinary Service Award

**DOI:** 10.1128/aac.00335-26

**Published:** 2026-04-01

**Authors:** Cesar A. Arias

**Affiliations:** 1 Houston Methodist Hospital 23534 , Houston, Texas, USA; 2 Houston Methodist Research Institute 167626 , Houston, Texas, USA; 3 Weill Cornell Medical College, Houston, Texas, USA

## EDITORIAL

*Antimicrobial Agents and Chemotherapy*’s high quality and timely publication of groundbreaking scientific research are critically dependent on the outstanding efforts of Editorial Board Members (https://journals.asm.org/journal/aac/board-editors) and *ad hoc* reviewers who generously contribute their time, wisdom, and talents to our journal.

In addition to thanking all of the reviewers who provide reviews each year, AAC has also developed an Extraordinary Service Award to recognize the efforts of our top reviewers. In this second year of the award, we recognize six awardees who were the top reviewers (by volume) in 2025 and were also all highly rated for review quality.

Yohei Doi, University of Pittsburgh School of Medicine

Gabriele Arcari, Universita degli Studi dell'Insubria

Andrea Endimiani, University of Bern

Jorge Arca-Suárez, Universitario de A Coruña (CHUAC), Sergas

Alejandro J. Vila, Instituto de Biologia Molecular y Celular de Rosario

Patricia A. Bradford, Antimicrobial Development Specialists LLC

AAC plans to continue this award each year not only to recognize the top contributors but also to elevate the importance of all the reviewers who help make AAC a great place to publish!

